# Electronic Health Records to Rapidly Assess Biosimilar Uptake: An Example Using Insulin Glargine in a Large U.S. Nursing Home Cohort

**DOI:** 10.3389/fphar.2022.855598

**Published:** 2022-05-04

**Authors:** Kaleen N. Hayes, Vincent Mor, Andrew R. Zullo

**Affiliations:** ^1^ Department of Health Services, Policy and Practice, Brown University School of Public Health, Providence, RI, United States; ^2^ Center of Innovation in Long-Term Services and Supports, Providence VA Medical Center, Providence, RI, United States; ^3^ Department of Epidemiology, Brown University School of Public Health, Providence, RI, United States

**Keywords:** health policy, pharmacoepidemiology, nursing homes, diabetes mellitus, insulin, big data, biological products

## Abstract

Large healthcare administrative databases, like Medicare claims, are a common means to evaluate drug policies. However, administrative data often have a lag time of months to years before they are available to researchers and decision-makers. Therefore, administrative data are not always ideal for timely policy evaluations. Other sources of data are needed to rapidly evaluate policy changes and inform subsequent studies that utilize large administrative data once available. An emerging area of interest in both pharmacoepidemiology and drug policy research that can benefit from rapid data availability is biosimilar uptake, due to the potential for substantial cost savings. To respond to the need for such a data source, we established a public-private partnership to create a near-real-time database of over 1,000 nursing homes’ electronic health records to describe and quantify the effects of recent policies related to COVID-19 and medications. In this article, we first describe the components and infrastructure used to create our EHR database. Then, we provide an example that illustrates the use of this database by describing the uptake of insulin glargine-yfgn, a new exchangeable biosimilar for insulin glargine, in US nursing homes. We also examine the uptake of all biosimilars in nursing homes before and after the onset of the COVID-19 pandemic. We conclude with potential directions for future research and database infrastructure.

## Introduction

Drug policies are critical to ensure that cost-effective and safe medications are used in clinical practice. Policies are most commonly implemented by payors and often take the form of formulary tiers and restrictions (Health Affairs, 2017) but may include direct mandates, incentive payments, and other mechanisms. Drug policies must be evaluated, often *via* pharmacoepidemiologic or economic studies, to ensure that the policies result in desired effects and do not have loopholes, inequities, or unintended consequences (European Monitoring Centre for Drugs and Drug Addiction, 2017).

A major area in drug policy is the use of biosimilars (Stern et al., 2021). Biologic medications make up approximately half of the expenditures for the top 25 drugs in the US and Canada (Tadrous et al., 2021; Tichy et al., 2021), with increasing costs each year. Thus, biosimilar use has the potential to result in substantial healthcare cost reductions. As of January 2022, 33 biosimilars have been approved for use in the US (U.S. Food & Drug Administration, 2021a), yet timely study of the uptake of biosimilars and related policies has been limited, especially for vulnerable populations like older adults and nursing home (NH) residents. Presently, some states allow biosimilars to be interchanged without notifying the prescriber or patient. Others require notification of the prescriber and/or patient. Yet others require that the prescriber specify that substitution is permissible (Cauchi, 2019). Thus, automatic substitution may not be consistently implemented across the US, but data are limited. A primary barrier to the timely evaluation of biosimilar uptake is a lag in the availability of healthcare administrative data (e.g., Medicare claims or national spending data). Rapid availability of data is critical to evaluate the uptake of biosimilars as they come to market and the impact of biosimilar-related policies on this uptake.

Insulin glargine-yfgn (Semglee®) was approved as an insulin product in the US on 11 June 2021 (U.S. Food & Drug Administration, 2021b), then as the first interchangeable biosimilar product for insulin glargine (Lantus®) on 28 July 2021 (U.S. Food & Drug Administration, 2021a; U.S. Food & Drug Administration, 2021c). Insulin glargine-yfgn could be directly prescribed as soon as it came to market if explicitly written for by the prescriber [similar to the other brand name insulin glargine (Basaglar®)]. However, after biosimilar approval insulin glargine-yfgn could be *directly interchanged* for insulin glargine (Lantus®) prescriptions at the point of dispensing by the pharmacy without prescriber approval, similar to the process of generic medication substitutions (U.S. Food & Drug Administration, 2017). Insulin biosimilars have the potential for substantial impact on healthcare cost savings; insulin glargine was the 3^rd^ top drug in expenditures (USD 9.7 billion) in the US in 2020 (Tichy et al., 2021). List price (without accounting for rebate programs, coupons, discounts, or wholesale pricing) for a 10 ml vial of insulin glargine-yfgn is USD 126 (GoodRx, 2022b); in contrast, the reference insulin glargine product is listed at USD 315 per vial (GoodRx, 2022a). Further, another biosimilar for insulin glargine (insulin glargine-aglr) was recently approved on 17 December 2021 (U.S. Food & Drug Administration, 2021a).

In US NHs alone, costs related to diabetes care exceeded $19.6 billion in 2012 (American Diabetes Association, 2013), with over one-third of residents having diabetes (Resnick et al., 2008; Dybicz et al., 2011; Newton et al., 2013). Fifty-nine percent of NH residents with diabetes are treated with insulin (Newton et al., 2013; Zullo et al., 2016a; Zullo et al., 2016b). Understanding early uptake of insulin biosimilars in NHs, where diabetes and insulin use are prevalent, may help to anticipate the trajectory of use in other clinical settings in the US while also providing due attention to a vulnerable population.

## Methods

### Overview of Data Sources

In mid-2020 we began a partnership to leverage data from 12 NH chains with a common EHR system (PointClickCare®) to answer stakeholder questions related to COVID-19 infection and vaccinations (White et al., 2020; Bardenheier et al., 2021a; White et al., 2021a; Bardenheier et al., 2021b; White et al., 2021b; White and Mor, 2021). These chains comprise more than 1,100 facilities located in almost every state in the contiguous US, with approximately 75,100 total beds. Residents of these facilities include both long-term NH residents as well as individuals undergoing post-acute care skilled nursing facility (SNF) stays. The residents of these chains are approximately representative of all individuals who reside in NH facilities nationally.

The NH EHR records contain information on the daily census (person-level file that contains each resident’s disposition on a given day, including transfers, discharges, and deaths), resident demographics (e.g., age, sex, race/ethnicity), nurses’ change in condition notes, immunization records, laboratory data, vital signs (e.g., blood pressure, temperatures) diagnosis codes, medication orders (medication initiation and discontinuation), non-medication orders (e.g., procedures, diagnostic testing, advance directives) and the electronic medication administration record (eMAR, contains an order number for each administration). In addition, the EHR contains Minimum Data Set (MDS) assessments, federally mandated clinical evaluations that must be conducted at admission for all NH residents and at least quarterly thereafter during their stay in the NH. Assessments include demographics, a 28-point scale of Activities of Daily Living (ADL) performance, a cognitive function assessment, and indicators of chronic comorbidities (e.g., diabetes). All datasets contain person-level information.

EHR data are transferred from PointClickCare® directly to our secure, encrypted server infrastructure. Access to the server network is highly controlled and internet access on the server is limited. Each component of the EHR (e.g., census, orders, eMAR, etc.) is transferred in a separate dataset for each chain and is only accessible in identifiable format by one data specialist. The data specialist then runs SAS programs to anonymize, clean, and process the data into SAS datasets, including macros to derive useful variables and “cross walks” to ensure data validity (e.g., the order identifier for a medication in the eMAR matches a corresponding order in the orders dataset). A chain-wide, anonymized patient identifier is generated based on each chain’s patient identifiers and can be used to link datasets. Brown University’s Institutional Review Board approved the study and waived the requirement for informed consent.

### Example: Insulin Glargine-Yfgn and General Biosimilar Uptake

We identified all NH residents (regardless of length of stay) with evidence of biosimilar use between 1 January 2018 and 30 November 2021 *via* medication orders in the EHR. A full list of eligible biosimilars is provided in Supplementary Table S1). Both generic and brand names for the biosimilar products were used to identify use. For the insulin glargine-yfgn analysis, we restricted this cohort to all individuals with use of insulin glargine-yfgn (defined as at least one record of administration in the eMAR) between 11 June 2021 (date of official approval by the FDA as an insulin product) and 30 November 2021 (the most recent data available at the time of analysis). We chose to define insulin glargine-yfgn use as at least one administration because we wanted to examine any uptake, rather than multiple administrations that represent a period of extended use. Nevertheless, we assessed the number of administrations of insulin glargine-yfgn for each resident to examine whether initial uptake was followed by sustained use. To understand the degree of use among all residents on any basal insulin regimen, we then calculated the monthly rate of residents with insulin glargine-yfgn use per 1,000 residents with any basal insulin use (intermediate-acting or long-acting insulins: insulin glargine [Lantus®, Basaglar®, or Semglee®], NPH, degludec, or detemir. To quantify changes over time, we divided the rate of residents with insulin glargine-yfgn use in November 2021 by the rate in June 2021 and estimated a parametric Wald 95% confidence interval (CI) for this value.

Next, we estimated the proportion of residents who switched from a non-glargine-yfgn basal insulin to insulin glargine-yfgn and, among these, time since first basal insulin use to glargine-yfgn use. We also calculated the time since admission to the NH to insulin glargine-yfgn initiation for all residents. Using lookback data in the 6 months prior to first insulin glargine-yfgn use for each resident, we described resident demographics and basic clinical characteristics using data from the MDS for residents with at least one MDS assessment of any type. We presented characteristics among all residents with insulin glargine-yfgn use and also stratified by whether the individual initiated insulin glargine-yfgn before versus after it was approved as an exchangeable biosimilar.

For the general biosimilar uptake analysis, we quantified the number of unique residents with biosimilar use for each month of the study. We graphed trends over time in all biosimilar use and use of insulin glargine-yfgn versus other biosimilars. We examined whether biosimilar use changed after the onset of the COVID-19 pandemic using an interrupted time series (ITS) analysis with segmented linear regression models. The ITS quantified the linear trend in the number of unique biosimilar users over time, the immediate effect of the onset of the pandemic (March 2020), and the effect of the onset of the pandemic on the linear trend in use over time. Because preliminary results showed a very little biosimilar use in 2018 and a drastic increase in biosimilar use when insulin glargine-yfgn came to market in June 2021, the ITS analysis used data from January 2019 to May 2021. Finally, we estimated the proportion of unique residents with orders for each type of biosimilar. SAS version 9.4 and STATA version 17 were used to conduct all analyses (SAS Institute, Cary, NC, United States; StataCorp, College Station, TX, United States).

## Results

### Insulin Glargine-Yfgn Uptake

**FIGURE 1:**
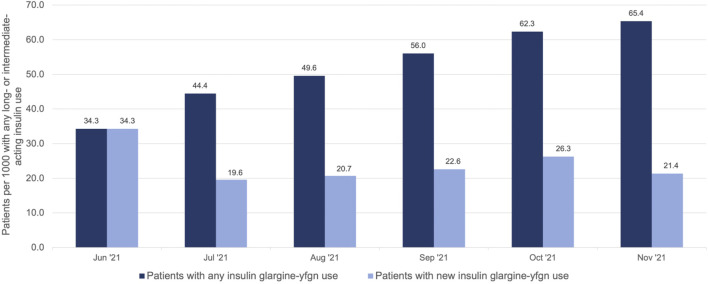
Number of nursing home residents with use of insulin glargine-yfgn (Semglee®) per 1,000 on any basal (long- or intermediate-acting) insulin use*, 2021. *includes insulin glargine [Lantus®*, Basaglar*®*, or Semglee*®]*, NPH, degludec, or detemir.*

We identified 1,567 unique NH residents who initiated insulin glargine-yfgn with 74,764 total recorded administrations in the eMAR. The median number of insulin glargine-yfgn administrations per resident was 27 (25th percentile: 12; 75th percentile: 60), and 97% of residents had more than one administration. In total, 1,554 (>99%) of these residents had at least one MDS assessment to provide characteristics. Residents initiating insulin glargine-yfgn had a median age of 68 years (25th percentile: 60; 75th percentile 76), 68% were White, 54% were male, and 54% switched from another basal insulin. The rate of individuals initiating insulin glargine-yfgn among all of those with any basal insulin use increased over time, from 34.3 residents using insulin glargine-yfgn for every 1,000 on basal insulin in June 2021 to 65.4 per 1,000 in November (Figure 1). This change over time represented a 1.90-fold increase (95% CI: 1.56–2.44). The rate of new initiators was greatest in June 2021 (34.3 per 1,000). Rates thereafter remained relatively stable, between approximately 20–26 new users per 1,000 on basal insulin. Among the 1,554 residents with an MDS assessment, 564 (37%) initiated insulin glargine-yfgn before biosimilar approval (Table 1). Compared to individuals whose first insulin glargine-yfgn use occurred after biosimilar approval, those with use prior had been in the NH for longer on average (median 17 versus 2 days) and more had use of another basal insulin prior to insulin glargine-yfgn (71% versus 44%). Age, sex, and race/ethnicity distributions were similar between groups. Supplementary Table S2 shows standardized mean differences that compare the distribution of characteristics between individuals starting insulin glargine-yfgn before versus after biosimilar approval.

**TABLE 1:** Characteristics of nursing home residents initiating insulin glargine-yfgn before versus after biosimilar approval, United States, 2018–2021.

	All Initiators^a^ (*N* = 1,554) n (%)^b^	Initiated prior to biosimilar approval (*N* = 564)^a^n (%)^b^	Initiated after biosimilar approval (N = 990)^a^n (%)^b^
Age, years (median [Q1, Q3])	68 (60, 76)	68 (60, 75)	68 (59, 76)
Male	833 (53.6)	311 (55.1)	522 (52.7)
Race/Ethnicity^c^			
White	1,051 (67.6)	382 (67.7)	669 (67.6)
Black	351 (22.6)	131 (23.2)	220 (22.2)
Hispanic	37 (2.4)	15 (2.7)	22 (2.2)
Asian, Pacific Islander, or Indigenous/Native American	16 (1.0)	8 (1.4)	8 (0.8)
Other/Missing	107 (6.9)	30 (5.3)	77 (7.8)
Time from first NH admission to first insulin glargine-yfgn use, days [median (Q1, Q3)]	2 (1, 35)	17 (1, 103)	2 (1, 9)
History of prior basal insulin use	833 (53.6)	401 (71.1)	432 (43.6)
Time since first basal insulin use, days (median [Q1, Q3])	33 (2, 401)	45 (7, 401)	15 (1, 397)
Renal impairment	282 (18.2)	144 (25.5)	138 (13.9)
Asthma or chronic obstructive pulmonary disease	220 (14.2)	117 (20.7)	103 (10.4)
Arrhythmias	127 (8.2)	73 (12.9)	54 (5.5)
Coronary artery disease	178 (11.5)	103 (18.3)	75 (7.6)
Dementia or Alzheimer’s disease	113 (7.3)	63 (11.2)	50 (5.1)
Diabetes	626 (40.3)	336 (59.6)	290 (29.3)
Heart failure	198 (12.7)	109 (19.3)	89 (9.0)
Hypertension	520 (33.5)	288 (51.1)	232 (23.4)
History of stroke or transient ischemic attack	100 (6.4)	51 (9.0)	49 (5.0)

Q1—25th percentile; Q3—75th percentile.

a With at least 1 MDS, Assessment of any type (admission, quarterly, or other); over 99% of all individuals with use.

b Unless otherwise indicated.

c Residents could be categorized into multiple race/ethnicity groups.

### General Biosimilar Use

**FIGURE 2:**
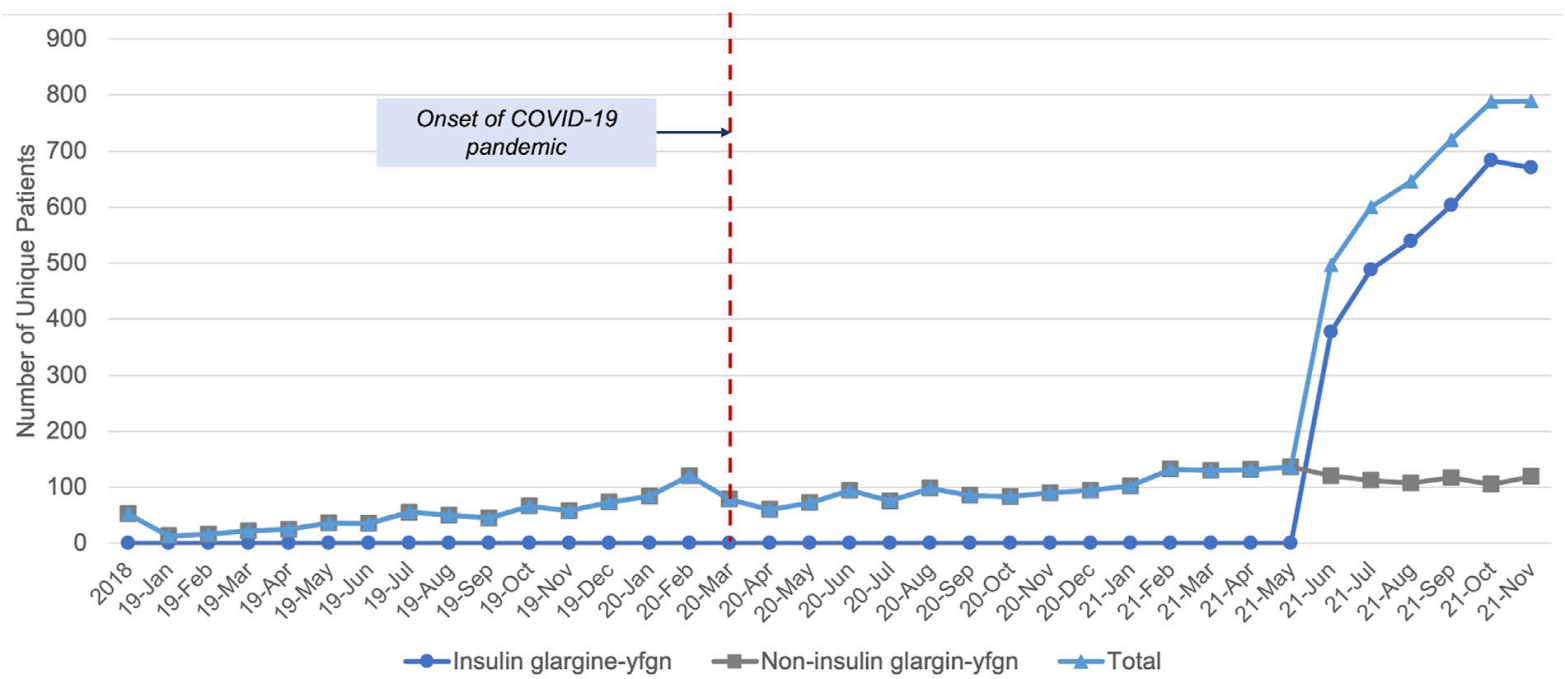
Nursing home residents with biosimilar use over time, 2018–2021.

Overall, the use of biosimilars was low (Figure 2). We identified 3,608 unique NH residents with biosimilar use. Less than 50 total individuals had any evidence of use in 2018. Residents on epoetin products made up the majority (52%), with insulin glargine-yfgn use as the second most common (43%). Filgrastim was the next most common (4.1% of residents), with all other biosimilars comprising less than 1% of use. The ITS analysis estimated a small but significant increase in the number of biosimilar users over time (average 7 [95% CI 5 to 9; *p* < 0.001] additional new users per month). The onset of the COVID-19 pandemic was associated with an immediate decrease in biosimilar use (effect of the pandemic: 38 fewer users [95% CI -57 to -18; *p* = 0.001]). However, the pandemic did not result in a significantly different trend in use over time leading up to when insulin glargine-yfgn came to market in June 2021 (interaction effect of pandemic*time: 2 fewer patients per month [95% CI -4 to 1; *p* = 0.32]).

## Discussion

We leveraged a first-of-its-kind, near-real-time database of EHR records to evaluate the uptake of biosimilars since 2018 among a large population of US NH residents. Biosimilar use decreased immediately at the onset of the COVID-19 pandemic. This finding is unsurprising given the extreme stresses placed on NH like outbreaks, staffing shortages, and competing clinical priorities combined with drug supply chain shortages. However, the trend in biosimilar use over time was not dramatically impacted by the pandemic. Further, the use of insulin glargine-yfgn appeared to increase over time. We observed an increase in the rate of insulin glargine biosimilar users from June to November 2021, with around one in 20 residents on basal insulin transitioning to glargine-yfgn by the end of the study period. Individuals with insulin glargine-yfgn use also made up 43% of all residents with biosimilar use, despite it being available for just 5 months of the 35-months study period.

This rapid uptake of insulin glargine-yfgn in NH compared to most other biosimilars may result from several concurrent phenomena. First, prescribers in NHs or the pharmacies that serve NHs may be encouraged to use biosimilars for medications administered during a post-acute care SNF stay. During post-acute stays, NHs receive bundled “per diem” payments for services provided, including medications, through the Medicare Part A SNF benefit (Congressional Research Service, 2016; Centers for Medicare and Medicaid Services, 2021). Cost-saving measures for drugs are therefore important in this period because the NH can incur a financial loss for the care provided if drugs consume too much or all of the fixed payment. Indeed, residents who started insulin glargine-yfgn after biosimilar approval had a median of 2 days in the facility (versus 17 in those starting before). These residents may have been on basal insulin prior to admission, and thus biosimilar use was a cost-reduction measure to continue this therapy during the SNF benefit period, rather than switching to a less appropriate but cheaper therapy like sliding-scale insulin (Munshi et al., 2016) or withholding the medication entirely.

Conversely, long-stay NH residents (generally defined as >100 days in-facility) have their medications covered *via* claims through Medicare Part D (Centers for Medicare and Medicaid Services, 2021). The Centers for Medicare and Medicaid Services has yet to implement substantive policies to encourage biosimilar use, so there is less financial incentive to use biosimilars among those covered through Part D. Potential policies may include specialty tier pricing or incentives (e.g., higher renumeration for the administration of biosimilar vs reference products). Indeed, limited drug reimbursements during SNF stays may serve as such an incentive in NHs, suggesting cost-savings for the provider or facility are a viable mechanism to increase biosimilar uptake. Future research should compare uptake of insulin biosimilars between the NH, community, and hospital settings.

Many other biologic therapies are administered less frequently than daily insulin (e.g., adalimumab, administered subcutaneously every 2 weeks (AbbVie Pharmaceuticals, 2018)). Thus, the low use of other biosimilars may result in part from use being unnecessary or delayed until discharge or the start of Part D coverage. In fact, epoetin and filgrastim biosimilars were the most common therapies used apart from insulin glargine-yfgn. Epoetin and filgrastim are administered up to three times per week and daily, respectively, suggesting that more frequently administered biologic therapies will have a higher uptake in NH due to required use during SNF stays. Insulin glargine-yfgn is available as a pen containing 300 units of insulin (Mylan Pharmaceuticals, 2021), so ease of administration may also increase its appeal. Finally, provider awareness may have been affected by targeted marketing efforts in NHs due to the large burden of diabetes among residents. Those who started insulin glargine-yfgn prior to its approval as a biosimilar were in-facility longer and had a higher prevalence of previous use of basal insulin; these individuals may have been long-stay residents that were switched by the prescriber from another basal insulin to insulin glargine-yfgn because of facility protocols or prescriber awareness. In contrast, those who initiated after biosimilar approval may have been started on the biosimilar version through automatic substitution by the long-term care pharmacy and were perhaps more likely to be short-stay (e.g., post-acute care SNF stay) residents.

The EHR data do have notable limitations. First, we are not able to consistently capture infusions or other medications administered *outside* the NH facility. Instead, we were required to rely on order fields that contained the medication names of interest and assumed the resident was receiving these treatments. Medication order fields for these drugs generally directed use at an outside provider (e.g., “Resident to visit Dr. X for infusion of rituximab-arrx”). Thus, uptake of biosimilars administered *via* infusions may be substantially greater than what we were able to observe. However, data were not limited by missing claims for Medicare Part B physician-administered medications as with traditional Medicare claims. Further, insulins are administered within the NH and thus are captured consistently in the eMAR. Second, we examined the uptake of insulin glargine-yfgn in a short period directly before and after biosimilar approval. Results are not necessarily representative of future prescribing behavior, which may be influenced by changes in practice and pricing developments. Third, unlike claims data, which are adjudicated by a payor and have been studied more for validity (e.g., studies validating diagnostic code algorithms), our data are generated in the course of usual care and have not yet been extensively validated. Further study should compare EHR and claims data to investigate whether there is substantial alignment in medication use as measured through these different sources. Finally, though our EHR database is, to our knowledge, the largest for a private-sector (i.e., non-Veterans Affairs) population of NH residents, a larger sample size will help to form better-powered studies of drug effects. Future work will expand these data to NH with other EHR vendors to increase sample size for pharmacoepidemiologic studies.

In conclusion, though stakeholder partnerships and infrastructure were required, we created a database of EHR information for NH residents. This database has proven valuable for rapid investigations during the COVID-19 pandemic; however, this detailed information is also well-suited for timely drug policy evaluations. For example, we illustrated that the biosimilar product insulin glargine-yfgn has had increasing uptake in NHs through 2021, potentially due to cost-savings for the NHs and parent NH companies. Future work could use these data or a similar data source to conduct pharmacoepidemiologic designs that take advantage of medication administration data, such as studies evaluating policies related to medication deprescribing.

## Data Availability

The data analyzed in this study was obtained from Brown University, the following licenses/restrictions apply: data use agreement. Requests to access these datasets should be directed to Kaleen Hayes, kaley_hayes@brown.edu.
