# Supplementary material for: Electronic Health Records to Rapidly Assess Biosimilar Uptake: An Example Using Insulin Glargine in a Large U.S. Nursing Home Cohort
Source: Front Pharmacol. 2022 May 4;13:855598. doi: 10.3389/fphar.2022.855598 (PMC9114471; doi:10.3389/fphar.2022.855598)
Supplement: Supplementary file 1 [file DataSheet1.docx]

**SUPPLEMENTAL TABLE 1. Complete list of reference products and corresponding eligible biosimilars approved in the U.S. as of December 1, 2021.**

| **Reference Product** | **Biosimilar Products** |
| --- | --- |
| adalimumab | adalimumab-fkjp  adalimumab-afzb  adalimumab-bwwd  adalimumab-adaz  adalimumab-adbm  adalimumab -atto |
| bevacizumab | bevacizumab-bvzr  bevacizumab-awwb |
| epoetin-alfa | epoetin alfa-epbx |
| etanercept | etanercept-ykro  etanercept-szzs |
| filgrastim | filgrastim-aafi  filgrastim-sndz |
| infliximab | infliximab-axxq  infliximab-qbtx  infliximab-abda  Infliximab-dyyb |
| pegfilgrastim | pegfilgrastim-apgf  pegfilgrastim-bmez  pegfilgrastim-cbqv  pegfilgrastim-jmdb |
| ranibizumab | ranibizumab-nuna |
| rituximab | rituximab-arrx  rituximab-pvvr  rituximab-abbs |
| trastuzumab | trastuzumab-anns  trastuzumab-qyyp  trastuzumab-dttb  trastuzumab-pkrb  trastuzumab-dkst |

Reference: US Food & Drug Administration. Purple Book Database of Licensed Biological Products. Published online December 30, 2021. Accessed January 3, 2022. https://purplebooksearch.fda.gov/

**SUPPLEMENTAL TABLE 2.** Characteristics of nursing home residents initiating insulin glargine-yfgn before versus after biosimilar approval, with standardized mean differences, United States, 2018-2021.

|  | **All Initiators***  **(N=1,554)**  **n (%)**** | **Initiated Prior to Biosimilar Approval**  **(N=564)**  **n (%)**** | **Initiated After Biosimilar Approval (N=990)**  **n (%)**** | **Standardized Mean Difference**^†^ |
| --- | --- | --- | --- | --- |
| Age, years (median, [Q1, Q3]) | 68 (60, 76) | 68 (60, 75) | 68 (59, 76) | 0.01 |
| Male | 833 (53.6) | 311 (55.1) | 522 (52.7) | 0.05 |
| Race/Ethnicity^+^ |  |  |  |  |
| White | 1051 (67.6) | 382 (67.7) | 669 (67.6) | 0.00 |
| Black | 351 (22.6) | 131 (23.2) | 220 (22.2) | 0.02 |
| Hispanic | 37 (2.4) | 15 (2.7) | 22 (2.2) | 0.03 |
| Asian, Pacific Islander, or Indigenous/Native  American | 16 (1.0) | 8 (1.4) | 8 (0.8) | 0.06 |
| Other/Missing | 107 (6.9) | 30 (5.3) | 77 (7.8) | 0.09 |
| Time from first NH admission to first insulin glargine-  yfgn use, days (median [Q1, Q3]) | 2 (1, 35) | 17 (1, 103) | 2 (1, 9) | 0.24 |
| History of prior basal insulin use | 833 (53.6) | 401 (71.1) | 432 (43.6) | 0.58 |
| Time since first basal insulin use, days (median  [Q1, Q3]) | 33 (2, 401) | 45 (7, 401) | 15 (1, 397) | 0.02 |
| Renal Impairment | 282 (18.2) | 144 (25.5) | 138 (13.9) | 0.29 |
| Asthma or Chronic Obstructive Pulmonary Disease | 220 (14.2) | 117 (20.7) | 103 (10.4) | 0.29 |
| Arrhythmias | 127 (8.2) | 73 (12.9) | 54 (5.5) | 0.26 |
| Coronary Artery Disease | 178 (11.5) | 103 (18.3) | 75 (7.6) | 0.32 |
| Dementia or Alzheimer’s Disease | 113 (7.3) | 63 (11.2) | 50 (5.1) | 0.23 |
| Diabetes | 626 (40.3) | 336 (59.6) | 290 (29.3) | 0.64 |
| Heart Failure | 198 (12.7) | 109 (19.3) | 89 (9.0) | 0.30 |
| Hypertension | 520 (33.5) | 288 (51.1) | 232 (23.4) | 0.60 |
| History of Stroke or Transient Ischemic Attack | 100 (6.4) | 51 (9.0) | 49 (5.0) | 0.16 |

*Q1 – 25^th^ percentile; Q3 – 75^th^ percentile.*

* with at least 1 MDS Assessment of any type (admission, quarterly, or other); over 99% of all individuals with use.

** Unless otherwise indicated.

^†^a standardized mean difference (SMD) >0.10 indicates a notable difference between groups.^23^ SMDs are calculated for mean values rather than median values as displayed.

^+^Residents could be categorized into multiple race/ethnic groups.
